# Pharmacists’ knowledge of medication errors and their attitudes toward reporting them in Saudi Arabia

**DOI:** 10.1097/MD.0000000000043035

**Published:** 2025-06-27

**Authors:** Abdullah Alahmari, Nehad Ahmed, Abdulaziz Alkheran, Abdullah Alotaibi, Moayad Alhamdani, Rashed Alhrbi, Sarah Fatani

**Affiliations:** a Department of Clinical Pharmacy, College of Pharmacy, Prince Sattam bin Abdulaziz University, Al Kharj, Saudi Arabia; b College of Pharmacy, Prince Sattam Bin Abdulaziz University, Al Kharj, Saudi Arabia; c Department of Clinical Pharmacy, College of Pharmacy, Taibah University, Medina, Saudi Arabia.

**Keywords:** attitudes, knowledge, medication errors, pharmacists, reporting

## Abstract

Medication errors result in negative outcomes such as increased mortality, morbidity, length of hospital stays and medical costs. Assessing pharmacists’ knowledge of medication errors and their attitudes toward reporting them is crucial for identifying gaps in awareness and practice. This study aims to evaluate pharmacists’ knowledge of medication errors and their attitudes toward reporting them in Saudi Arabia. The present study was a cross-sectional study that included an online survey. Descriptive statistical analysis was conducted for all study variables, and linear regression analyses were performed to identify factors influencing pharmacists’ knowledge of medication errors and their attitudes toward reporting them. The analysis was carried out using Jamovi software. A total of 527 pharmacists completed the survey. Over half of the participants were male (52.37%), and 59.39% were between the ages of 20 and 25. Over 25% of the pharmacists worked in hospitals, and 22.39% were employed in community pharmacies. Additionally, more than 56% of pharmacists have 8 or more years of work experience. The majority of pharmacists (90.13%) had limited knowledge about medication errors, but they maintained a positive attitude toward reporting these errors (96.39%). Pharmacists’ attitudes toward medication error reporting were not significantly associated with the pharmacists’ demographic data. However, pharmacists’ knowledge of medication errors was significantly related to gender and age. To enhance medication safety and minimize errors, targeted educational programs should be introduced, with an emphasis on improving pharmacists’ knowledge. Workplace-specific interventions are essential, alongside fostering a “just culture” to encourage transparent reporting without fear of blame. Policymakers and institutions must strengthen medication safety protocols, implement digital reporting tools, and promote interprofessional collaboration to reduce preventable errors and improve patient care.

## 1. Introduction

The World Health Organization started the “Medication without Harm” campaign in 2017 as a global initiative to improve patient safety by strengthening healthcare systems and reducing drug errors.^[1]^ Medication errors can occur at any level of the medication-use process; thus, countries must take action to address and improve medication-use practices by healthcare providers.^[2]^ Patient safety has always been the priority for all healthcare providers to provide high-quality care. The increased use of technology, as well as the involvement of a wide range of health professionals, has resulted in a complicated healthcare delivery system.

The National Coordinating Council for Medication Error Reporting and Prevention (NCC MERP) defines a medication error as any preventable incident that may cause or lead to inappropriate pharmaceutical usage or patient harm when the medication is in the hands of a healthcare practitioner, patient, or consumer.^[3]^ Such events may be associated with professional practice, healthcare items, methods, and systems, such as prescribing, order communication, product labeling, packaging, nomenclature, compounding, dispensing, distribution, administration, education, monitoring, and use.^[3]^ The literature on medication mistake prevention is developing, showing that these occurrences can occur at any stage of the medication process and in a variety of healthcare settings, including general hospitals, primary care, senior acute care, and pediatric institutions.^[4–11]^

Medication errors are typically connected with the patient, health care provider, or medication, and the primary factors contributing to the occurrence of medication errors are the health care facility, method of drug administration, and clinical scenario. In general, medication errors result in negative outcomes such as increased mortality, morbidity, length of hospital stay, and medical costs.^[12]^ In the United States of America, 7000 deaths are reported in hospitals each year as a result of pharmaceutical errors. Medication mistakes are expected to cost the United Kingdom National Health Service around £1.1 billion each year. As a result, in the contemporary population, medication errors constitute a critical patient safety concern that must be monitored and addressed.^[13]^

Many countries have implemented techniques to address safety issues that may contribute to drug errors, such as intervention tactics and reporting systems. In the United States, there are several reporting systems accessible, including the Food and Drug Administration’s MedWatch Reporting Program, the United States Pharmacopeia-Institute for Safe Medication Practices’ Medication Errors Reporting Program, and the MedMarx® Reporting Program.^[14]^ The European Medicines Agency coordinates the EU’s pharmacovigilance network among member states. Medication mistakes are reported to national pharmacovigilance systems to EudraVigilance, the EU’s adverse reaction database.^[15]^ In poor nations such as the Middle East, pharmacovigilance systems are implemented and used differently. Data on drug error reporting is primarily from hospital and primary care research.^[16,17]^

The Saudi context is particularly relevant for this study due to the country’s rapidly evolving healthcare system, increasing pharmacist workforce, and ongoing efforts to enhance medication safety. With a high burden of chronic diseases and expanding healthcare access, understanding pharmacists’ knowledge and attitudes toward medication error reporting is critical for improving patient outcomes. Additionally, cultural and systemic factors in Saudi Arabia – such as hierarchical structures in healthcare and underreporting tendencies – may influence error-reporting behaviors, making this study essential for guiding localized interventions.

Medication errors are a major cause of patient harm globally, including in Saudi Arabia, where they can occur at any stage of the medication process – from prescribing to dispensing and administration. These errors can have significant impacts on patient health. Assessing pharmacists’ knowledge of medication errors and their attitudes toward reporting them is crucial for identifying gaps in awareness and practice. This insight can help develop more effective strategies for preventing errors and enhancing patient safety. This study aims to evaluate pharmacists’ knowledge of medication errors and their attitudes toward reporting them in Saudi Arabia.

## 2. Methods

A cross-sectional study was carried out to assess pharmacists’ knowledge of medication errors and their attitudes toward reporting them in Saudi Arabia. The questionnaire used in this study had been developed and validated in prior studies.^[2,18]^ It was administered via a web-based survey to enable nationwide participation among pharmacists. The survey was conducted over 3 months, from August to October 2024, ensuring sufficient time for data collection and analysis.

The sample size was calculated using the Rao sample size calculator, with parameters including a population size of 20,000, a margin of error of 5%, a confidence level of 95%, and an assumed response distribution of 50%. Based on these parameters, the minimum recommended number of respondents for the survey was determined to be 377.

All pharmacists were assured of anonymity and confidentiality. The questionnaire was divided into 3 sections. The first section collected socio-demographic information, such as gender, age, years of experience, work setting, and education level. The second section contained 10 questions regarding knowledge of medication errors, while the third section included 6 questions assessing attitudes toward reporting medication errors. Knowledge and attitudes were evaluated using a 2-point Likert scale (agree or disagree). Data was gathered through a web-based survey via Google Forms, with the survey link shared with pharmacists via WhatsApp.

Descriptive statistical analysis was conducted for all study variables, and linear regression analyses were performed to identify factors influencing pharmacists’ knowledge of medication errors and their attitudes toward reporting them. Regarding knowledge assessment, correct answers were assigned a score of 1, while incorrect answers received a score of 0. The average knowledge scores were then calculated. A score below 50% was considered poor knowledge, between 50% and 75% indicated moderate knowledge, and above 75% represented good knowledge. For the attitude assessment, responses were categorized into 2 groups: “agree” was given a score of 1, and “disagree” a score of 0. The average attitude scores were calculated based on this. An attitude score below 50% reflected a negative attitude, while a score of 50% or higher indicated a positive attitude.^[19]^ The analysis was carried out using Jamovi software.

## 3. Results

A total of 527 pharmacists completed the survey. Over half of the participants were male (52.37%), and 59.39% were between the ages of 20 and 25. More than 72% of the pharmacists held a bachelor’s degree, while 27.70% had a master’s or doctorate. Over 25% of the pharmacists worked in hospitals, and 22.39% were employed in community pharmacies. Additionally, more than 56% of the pharmacists had 8 or more years of work experience (Table 1).

**Table 1:** The socio-demographic characteristics of respondents (n = 527).

Variable	Category	Number	Percentage
Gender	Male	276	52.37
	Female	251	47.63
Age	20–25	313	59.39
	25–30	168	31.88
	30–35	35	6.64
	35–40	6	1.14
	>40	5	0.95
Highest education	Undergraduate	381	72.30
	Postgraduate	146	27.70
Work setting	Hospital	136	25.81
	Community pharmacy	118	22.39
	Others*	273	51.80
Working experience	<8 yr	231	43.83
	More than 8 yr	296	56.17

* Others: health centers, company, university.

Only 24.86% of pharmacists acknowledged that medication errors happen when a pharmacist fails to ensure the correct patient receives the proper medication, dosage, and quantity. Meanwhile, 23.91% agreed that such errors occur when medication labels are of poor quality or damaged. Around 27% of pharmacists recognized that medication errors can result from incorrect dosage calculations, and 27.70% agreed that distractions from other patients or coworkers can lead to errors. Only 21.06% of pharmacists believed that errors arise when a pharmacist provides unclear or incorrect instructions during medication dispensing, while 28.46% felt that pharmacist fatigue contributes to medication errors (Table 2).

**Table 2:** Pharmacists’ knowledge of medication errors.

Variable	Category	Male	Female	Total
A medication error occurs when a pharmacist dispenses the incorrect drug, dose, quantity, or patient	Yes	71 (25.72)	60 (23.90)	131 (24.86)
	No	205 (74.28)	191 (76.10)	396 (75.14)
A medication error can happen if the doctor’s handwriting on the prescription is unclear or hard to read	Yes	82 (29.71)	74 (29.48)	156 (29.60)
	No	194 (70.29)	177 (70.52)	371 (70.40)
Poor-quality or damaged medication labels may lead to errors	Yes	65 (23.55)	61 (24.30)	126 (23.91)
	No	211 (76.45)	190 (75.70)	401 (76.09)
Medication errors can happen when drug names look or sound alike, causing confusion	Yes	73 (26.45)	68 (27.09)	141 (26.76)
	No	203 (73.55)	183 (72.91)	386 (73.24)
A medication error occurs when a doctor prescribes the incorrect dose	Yes	87 (31.52)	57 (22.71)	144 (27.32)
	No	189 (68.48)	194 (77.29)	383 (72.68)
Pharmacists may cause medication errors if they make mistakes in dose calculations	Yes	72 (26.09)	68 (27.09)	140 (26.57)
	No	204 (73.91)	183 (72.91)	387 (73.43)
A medication error occurs when a pharmacist dispenses the wrong medication	Yes	73 (26.45)	67 (26.69)	140 (26.57)
	No	203 (73.55)	184 (73.31)	387 (73.43)
A medication error occurs when a pharmacist provides incorrect or unclear instructions to a patient	Yes	56 (20.29)	55 (21.91)	111 (21.06)
	No	220 (79.71)	196 (78.09)	416 (78.94)
When pharmacists are interrupted by others, mistakes in dispensing can happen	Yes	84 (30.43)	62 (24.70)	146 (27.70)
	No	192 (69.57)	189 (75.30)	381 (72.30)
Pharmacist exhaustion increases the risk of medication errors	Yes	80 (28.99)	70 (27.89)	150 (28.46)
	No	196 (71.01)	181 (72.11)	377 (71.54)

Table 3 illustrates pharmacists’ attitudes toward medication error reporting. Approximately 27% of pharmacists reported that they did not report medication errors due to fear of their superior’s reaction, while 27.89% said that most of their colleagues ignored reporting. Around 24% of pharmacists mentioned that they did not report errors because they felt the issue was not serious enough to warrant reporting. Additionally, 25.81% of pharmacists stated they failed to report medication errors because the error did not affect or harm the patient.

**Table 3:** Attitude toward medication error reporting.

Variable	Category	Male	Female	Total
I failed to report the medication error because I was afraid of the reaction from my superior	Agree	65 (23.55)	75 (29.88)	140 (26.57)
	Disagree	211 (76.45)	176 (70.12)	387 (73.43)
I failed to report the medication error because I was afraid of the reaction received from my coworkers	Agree	61 (22.10)	59 (23.51)	120 (22.77)
	Disagree	215 (77.90)	192 (76.49)	407 (77.23)
I failed to report the medication error because most of my colleagues ignored the reporting	Agree	69 (25.00)	78 (31.08)	147 (27.89)
	Disagree	207 (75.00)	173 (68.92)	380 (72.11)
I failed to report the medication error because I thought the error was not serious enough to warrant reporting	Agree	64 (23.19)	63 (25.10)	127 (24.10)
	Disagree	212 (76.81)	188 (74.90)	400 (75.90)
I failed to report the medication error because I’m afraid that I might lose the job	Agree	71 (25.72)	52 (20.72)	123 (23.34)
	Disagree	205 (74.28)	199 (79.28)	404 (76.66)
I failed to report the medication error because the error did not reach or harm the patient	Agree	76 (27.54)	60 (23.90)	136 (25.81)
	Disagree	200 (72.46)	191 (76.10)	391 (74.19)

Correct answers in the knowledge assessment were scored 1, and incorrect answers scored 0, with scores below 50% indicating poor knowledge, 50% to 75% for moderate knowledge, and above 75% indicating good knowledge. In the attitude assessment, responses were scored 0 for “agree” and 1 for “disagree,” with scores below 50% indicating a negative attitude and scores of 50% or higher indicating a positive attitude. Although the majority of pharmacists (90.13%) had limited knowledge about medication errors, they maintained a positive attitude toward reporting these errors (96.39%) (Table 4).

**Table 4:** Knowledge and attitude scores.

Knowledge score	Male	Female	Total
Poor knowledge (<50%)	252 (91.30)	223 (88.84)	475 (90.13)
Moderate knowledge (50%–75%)	20 (7.25)	22 (8.77)	42 (7.97)
Good knowledge (>75%)	4 (1.45)	6 (2.39)	10 (1.90)
Average knowledge score = 26.28% (average knowledge score was 26.92 for males and 25.58 for females)			

Attitude score	Male	Female	Total
Negative attitude (<50%)	7 (2.54)	12 (4.78)	19 (3.61)
Positive attitude (>50%)	269 (97.46)	239 (95.22)	508 (96.39)
Average attitude score = 74.92 (average attitude score was 75.48 for males and 74.30 for females)			

Table 5 shows the relationships between personal data and pharmacists’ knowledge and attitudes. Pharmacists’ attitudes toward medication error reporting showed no significant link with demographic factors. However, their knowledge of medication errors varied by gender and age: male pharmacists demonstrated significantly higher knowledge than their female counterparts (*P* < .001), and those over 25 years old scored higher than younger pharmacists (*P* = .046). The observed differences in pharmacists’ knowledge of medication errors – where male pharmacists and those over 25 scored significantly higher – may stem from variations in training exposure, work experience, or confidence in self-reporting. Older pharmacists likely benefit from accumulated clinical experience, while gender disparities could reflect differences in educational emphasis, workplace roles, or even response bias in self-assessed knowledge. However, since demographic factors did not influence error-reporting attitudes, systemic interventions – such as standardized training programs and mentorship for younger and female pharmacists – could help bridge these knowledge gaps while maintaining high reporting willingness across all groups.

**Table 5:** The associations between personal data and pharmacists’ knowledge and attitude.

Personal data	*P*-value (knowledge)	*P*-value (attitude)
Gender	**<.001**	.451
Age	**.046**	.646
Education level	.279	.061
Work setting	.202	.539
Working experience	.437	.897

## 4. Discussion

Medication errors are preventable incidents that can harm patients while prescribing, dispensing, or administering medications. These errors can occur at various stages of the medication process and are a significant concern across healthcare settings worldwide. They represent a major patient safety issue, potentially leading to adverse drug reactions, extended hospital stays, additional treatments, or even death. Pharmacists play a key role in preventing medication errors by ensuring proper medication management and providing patient counseling. This study aimed to assess pharmacists’ knowledge of medication errors and their attitudes toward reporting them in Saudi Arabia. The results indicate that pharmacists had a limited understanding of medication errors but displayed a positive attitude toward both recognizing and reporting them. Factors such as gender and age were found to influence pharmacists’ knowledge of medication errors.

The current study found that pharmacists had insufficient knowledge about medication errors. Samsiah et al found that 41.2% of healthcare practitioners in their study had a good understanding of medication errors and reporting.^[20]^ Rickles et al noted that nearly 80% of pharmacy students had not received training in communicating medication errors, stressing the importance of additional training in this area. They recommended the development of educational interventions to provide consistent instruction on effective communication regarding medication errors.^[21]^ In contrast, Mamat et al reported that while pharmacists had good knowledge of medication errors, their attitude toward reporting these errors remained poor.^[2]^

Abdel-Latif pointed out disparities in healthcare professionals’ awareness of medication errors in hospitals, noting that the knowledge gaps highlight the critical need for initiatives to improve understanding of this issue in Saudi hospitals.^[22]^ Supporting this, Alsulami et al found that 72% of healthcare practitioners in a Saudi tertiary care setting were unaware of proper medication error-reporting procedures.^[23]^ Similarly, Alanzi et al reported that while healthcare providers in Saudi Arabia scored an average of 77% on knowledge about medication errors, their attitudes toward the issue averaged 72%, indicating room for improvement.^[24]^ Additionally, Alshammari et al identified significant deficiencies in medication error reporting, knowledge of error stages, and training among Saudi healthcare professionals, emphasizing an urgent need for corrective measures.^[25]^

The current study found that pharmacists have a positive attitude toward reporting medication errors. Carandang et al reported that the majority of healthcare practitioners (58%) exhibited unfavorable attitudes toward medication error reporting, with only pharmacists showing a higher proportion (52%) of respondents with favorable attitudes.^[26]^ Furthermore, Mamat et al found that while 90% of pharmacists in their study demonstrated good knowledge of medication errors, only 25.4% had a favorable attitude toward reporting these errors.^[2]^ Pharmacists are committed to ensuring patient safety and well-being, understanding that reporting medication errors is essential for identifying risks and preventing future harm. This positive attitude toward reporting is often driven by a strong sense of professional responsibility to enhance patient outcomes.

The current study found no significant association between pharmacists’ attitudes toward medication error reporting and their demographic data. However, pharmacists’ knowledge of medication errors was significantly related to gender and age, with male pharmacists demonstrating greater knowledge than female pharmacists, and those over 25 years of age having more knowledge than those younger than 25. Mamat et al reported that knowledge was not associated with demographic data, but they found that female pharmacists (*P* = .001), more experienced pharmacists (*P* = .012), and those working in primary health clinics (*P* = .014) had a more favorable attitude toward reporting medication errors.^[2]^ Carandang et al stated that while health practitioners’ knowledge of medication error reporting was significantly related to their age, their attitudes and practices toward reporting were not affected by age. Additionally, the gender, work environment, and years of experience of healthcare practitioners showed no significant relationship with their knowledge or attitudes toward medication error reporting.^[26]^ These findings suggest that age and gender may play significant roles in shaping pharmacists’ knowledge of medication errors, with older and male pharmacists potentially having more exposure to error-related training and experiences. However, these results may also highlight the need for targeted educational interventions to address knowledge gaps across different groups.

Ther are several limitations for the study. Pharmacists may provide answers that they believe are socially acceptable or expected, such as over-reporting their knowledge of medication errors or their willingness to report them, to avoid being perceived negatively. Furthermore, online surveys might only reach pharmacists with internet access and digital literacy, leaving out those less comfortable with technology or without reliable connectivity. Additionally, as an online survey lacks the opportunity for direct follow-up or clarification, participants may misunderstand questions or fail to provide sufficient context for their answers, which can impact the validity of the data.

## 5. Conclusion

This study highlights important insights into pharmacists’ knowledge and attitudes regarding medication errors. Despite the pharmacists’ positive attitude towards reporting medication errors, we found their overall knowledge of medication errors to be insufficient. These findings suggest that while pharmacists are willing to engage in error reporting, there are gaps in their understanding of medication errors that need to be addressed. To enhance medication safety and minimize errors, targeted educational programs should be introduced, with an emphasis on improving pharmacists’ knowledge. Targeted educational programs should include mandatory medication safety training in continuing education, using workshops, e-learning, and case-based learning. Hospitals and pharmacies can conduct regular in-house sessions, while universities and professional bodies standardize up-to-date content. Incentives like CPD credits can boost participation, ensuring pharmacists gain practical skills to improve medication safety.

## Acknowledgments

The authors extend their appreciation to Prince Sattam Bin Abdulaziz University for funding this research work through the project number (PSAU/2024/03/29866).

## Author contributions

**Conceptualization:** Nehad Ahmed.

**Data curation:** Abdulaziz Alkheran, Abdullah Alotaibi, Moayad Alhamdani, Rashed Alhrbi.

**Formal analysis:** Abdullah Alahmari.

**Funding acquisition:** Abdullah Alahmari.

**Methodology:** Abdullah Alahmari.

**Software:** Sarah Fatani.

**Supervision:** Sarah Fatani.

**Writing – original draft:** Nehad Ahmed.

**Writing – review & editing:** Abdullah Alahmari.
